# The Use of Different International References to Assess Child Anthropometric Status in a Malaysian Population

**DOI:** 10.1016/j.jpeds.2017.07.049

**Published:** 2017-11

**Authors:** Uttara Partap, Elizabeth H. Young, Pascale Allotey, Manjinder S. Sandhu, Daniel D. Reidpath

**Affiliations:** 1 Department of Medicine, University of Cambridge, Cambridge, UK; 2 Wellcome Trust Sanger Institute, Hinxton, United Kingdom; 3 Jeffrey Cheah School of Medicine and Health Sciences, Monash University Malaysia, Selangor, Malaysia; 4 South East Asia Community Observatory, Segamat, Malaysia

**Keywords:** underweight, overweight, obesity, double burden of malnutrition, BMI, Body mass index, CDC, Centers for Disease Control and Prevention, IOTF, International Obesity Task Force, WHO, World Health Organization, SEACO, South East Asia Community Observatory

## Abstract

**Objective:**

To assess the prevalence of child underweight, overweight, and obesity in a Malaysian population according to 3 international references because classification of anthropometric status may differ according to the reference used to express body mass index (BMI).

**Study design:**

We assessed data from 6414 children aged 6-18 years, collected by the South East Asia Community Observatory. Child underweight, overweight, and obesity were expressed according to 3 internationally used BMI references: World Health Organization 2007, International Obesity Task Force 2012, and Centers for Disease Control and Prevention 2000. We assessed agreement in classification of anthropometric status among the references using Cohen's kappa statistic and estimated underweight, overweight, and obesity prevalence according to each reference using mixed effects Poisson regression.

**Results:**

There was poor to moderate agreement between references when classifying underweight, but generally good agreement when classifying overweight and obesity. Underweight, overweight, and obesity prevalence estimates generated using the 3 references were notably inconsistent. Overweight and obesity prevalence estimates were higher using the World Health Organization reference vs the other 2, and underweight prevalence was up to 8.5% higher and obesity prevalence was about 4% lower when using the International Obesity Task Force reference.

**Conclusions:**

The choice of reference to express BMI may influence conclusions about child anthropometric status and malnutrition prevalence. This has implications regarding strategies for clinical management and public health interventions.

In many middle income populations, including those in Asia, the prevalence of child overweight and obesity is increasing even though the burden of child underweight remains high.^1^, ^2^ Within Asia, Malaysia has a particularly high prevalence of child overweight and obesity, with recent evidence also indicating a notable prevalence of underweight.^1^, ^3^, ^4^, ^5^, ^6^, ^7^ This double burden of malnutrition may be understood within the context of epidemiologic transitions, whereby changes in lifestyle habits are leading to a positive shift in body mass index (BMI) distributions in populations that used to be predominantly normal or underweight.^2^, ^8^ Child underweight and overweight are associated with distinct adverse consequences in childhood, and both are understood to contribute to the development of cardiometabolic diseases in adulthood.^9^, ^10^, ^11^, ^12^, ^13^ Given these far-reaching public health consequences, it is essential to identify and address both underweight and overweight among children, especially in nations such as Malaysia where there is evidence that both types of malnutrition coexist.

Although the World Health Organization (WHO) 2006 standards are widely accepted as ideal for assessing anthropometric status among children aged 0-5 years,^14^ there is no single, universal reference or standard for children of older ages. Apart from locally constructed references, any 1 of 3 international references is typically used in research or recommended in local clinical practice guidelines: the WHO 2007 reference for children aged 5-19 years, the International Obesity Task Force (IOTF) 2012 reference, and the Centers for Disease Control and Prevention (CDC) 2000 reference. These differ in terms of the populations and methods used to develop BMI-for-age reference curves, and in terms of their recommended cut-offs.^15^, ^16^, ^17^, ^18^ Research comprehensively exploring the comparability of each of these 3 references against the other in classifying both types of malnutrition is limited, especially within Asia.^19^, ^20^, ^21^, ^22^, ^23^ The consistency in their classification of child anthropometric status and malnutrition prevalence may have implications in terms of clinical management and health resource planning in Asian populations. We, therefore, aimed to examine the comparability of the WHO 2007, IOTF, and CDC references in assessing anthropometric status in a general population of children aged 6-18 years in Malaysia.

## Methods

We used data from a population-wide health survey conducted from 2013 to 2014 by the South East Asia Community Observatory (SEACO), a health and demographic surveillance system in southern peninsular Malaysia.^24^ SEACO conducts annual enumerations in 5 subdistricts of the Segamat district in Johor state, and in addition, acts as a platform for focused health surveys and studies. Ethical approval for all data collections undertaken within the SEACO health and demographic surveillance system are obtained from the Monash University Human Research Ethics Committee, and informed consent is obtained before data collection from all participants and their parents or guardians where relevant.

All data for the SEACO health survey were collected on encrypted tablets. For this study, we used information collected from children aged 6-18 years on height and weight, sex, age, and ethnicity. Height and weight measurements were taken by trained staff following standardized procedures in accordance with the WHO STEPwise approach to Surveillance manual,^25^ using Transtek digital weighing scales with height gauges (model GBS-721; Zhongshan Transtek Electronics, Zhongshan, China); instruments used were calibrated before and after the survey. Measurements were taken with participants wearing light clothing, no shoes, and no headgear where possible and appropriate.

### Statistical Analyses

Children's anthropometric status (underweight, normal weight, overweight, or obese) was expressed using the Zanthro package in Stata (developers: Suzanna I. Vidmar, Royal Children's Hospital, Melbourne, Australia; Tim J. Cole, University College London, London, UK; Huiqi Pan, University College London, London, UK) according to 3 BMI references, described below.^26^

The WHO 2007 reference, for children aged 5-19 years, is based upon information on 22 917 children from 3 national surveys conducted in the US from 1963 to 1974, with additional data from the WHO Multicenter Growth Reference Study (1997-2003). Underweight, overweight, and obese are defined as BMI-for-age less than 2 SDs below the mean, greater than 1 SD above the mean, and greater than 2 SDs above the mean respectively.^16^, ^27^

The CDC 2000 reference, for children aged 2-20 years, is based on the US surveys described above, with additional data from further national surveys conducted up until 1994 (total sample size: 32 653). Underweight, overweight, and obesity are defined as BMI-for-age less than the fifth percentile, greater than or equal to the 85th percentile and greater than or equal to the 95th percentile, respectively. To note, the CDC terms the latter 2 cut-offs as “at risk of overweight” and “overweight” respectively.^17^, ^28^

The IOTF 2012 reference was constructed from national surveys undertaken from 1963 to 1993, in 192 727 children aged 2-18 years from 6 countries (Singapore, Hong Kong, Great Britain, The Netherlands, the US, and Brazil). The reference provides percentile cut-offs corresponding to a BMI of 18.5, 25, and 30 kg/m^2^ at 18 years of age for underweight, overweight, and obesity, respectively. Thus, underweight is defined as BMI-for-age less than the 15.5th percentile in boys and the 16.5th percentile in girls, overweight as BMI-for-age greater than or equal to the 90.5th percentile in boys and the 89.3rd percentile in girls, and obesity as BMI-for-age greater than or equal to the 98.9th percentile in boys and the 98.6th percentile in girls.^15^

To allow for comparability across references, we used data on children aged 6-18 years. We initially used the Cohen kappa statistic to assess the overall agreement between the 3 references when classifying underweight, overweight, or obesity. Kappa <0.6 was defined as poor agreement, 0.6 to <0.8 as moderate agreement, 0.8 to <0.9 as good agreement and ≥0.9 as excellent agreement.^29^ To explore whether these differed by population subgroups, we additionally explored agreement across categories of sex, age, and ethnicity. Following this, for each BMI reference, we used mixed effects Poisson regression models to estimate the prevalence of underweight, overweight, and obesity overall and across categories of sex, age, and ethnicity. All regression models were based on analysis of complete records and were adjusted for clustering at the household level.

Analyses were conducted using Stata 13 and 14 (StataCorp, College Station, Texas).

## Results

The analyses included 6414 children aged 6-18 years, approximately one-half of whom were boys. The majority of children were of Malay ethnicity, followed by Chinese and Indian ethnicity. There were no differences by sex in the distribution of children across categories of ethnicity and age (Table I).

**Table I:** Demographic characteristics of study population

	Overall	Male	Female	*P*
N (%)	6414 (100.0)	3174 (49.5)	3240 (50.5)	
Age (y)				
6-9	1772 (27.6)	899 (28.3)	873 (26.9)	
10-14	2519 (39.3)	1257 (39.6)	1262 (39.0)	
15-19	2123 (33.1)	1018 (32.1)	1105 (34.1)	.19
Ethnicity, n (%)				
Malay	4317 (67.3)	2131 (67.1)	2186 (67.5)	
Indian	632 (9.9)	322 (10.1)	310 (9.6)	
Chinese	1272 (19.8)	625 (19.7)	647 (20.0)	
Bumiputera/Orang Asli	116 (1.8)	61 (1.9)	55 (1.7)	
Other	77 (1.2)	35 (1.1)	42 (1.3)	.81

Differences in distributions across categories between male and female subjects were compared using the Pearson χ^2^ test.

We initially assessed agreement in classification of anthropometric status between the WHO, IOTF, and CDC references (Tables II and III; available at www.jpeds.com). There was poor to moderate agreement between the 3 references when classifying child underweight, with poorest agreement between the WHO and IOTF references. Between the WHO and IOTF references, agreement was particularly poor among girls, children aged 15-18 years, and those of other ethnicity. When classifying child overweight, there was good to excellent agreement between the references, especially between the IOTF and CDC references. Agreement was similar when examining child obesity, particularly between the WHO and CDC references, overall and across population subgroups. Agreement between the other references was more variable across subgroups (Table II).

We then examined estimates for underweight, overweight, and obesity prevalence obtained using the 3 references. Overall, there was a notable prevalence of underweight, and a high prevalence of overweight and obesity in this population, according to all 3 references. Estimates for underweight prevalence varied widely between the 3 references. Estimates for overweight prevalence were similar when using the IOTF and the CDC references, but estimates using the WHO reference were markedly higher. Obesity prevalence was also highest when using the WHO reference and comparable with estimates using the CDC reference, while prevalence estimates using the IOTF reference were notably lower (Figure; available at www.jpeds.com, Table IV, Table V, Table VI.

We also observed differences in the distribution of child underweight across population subgroups when using the 3 references. Although underweight prevalence was marginally higher among boys vs girls when using the WHO and CDC references, it was notably lower among boys when using the IOTF reference. When using the WHO reference, underweight prevalence was slightly lower among children aged 10-14 and 15-19 years vs those aged 6-9 years, but when using both IOTF and CDC references, it was markedly lower in children aged 10-14 years (Table IV). In contrast, there was greater consistency in the distribution of overweight and obesity across population subgroups when using any of the 3 references. The prevalence of overweight and obesity was highest among boys and children of Chinese ethnicity, and lowest among children aged 15-18 years and children of Indian ethnicity (Tables V and VI).

**Table IV:** Prevalence of underweight among children in Segamat, Malaysia according to 3 international BMI references

	Prevalence (95% CI)		
	WHO	IOTF	CDC
Overall	4.6 (3.7, 5.5)	13.1 (11.8, 14.4)	7.9 (6.8, 9.0)
Sex, n (%)			
Male	5.5 (3.7, 7.3)	12.7 (11.5, 14.0)	9.4 (8.4, 10.5)
Female	4.3 (2.9, 5.6)*	16.1 (14.7, 17.5)	7.9 (6.2, 9.5)
Age, y, n (%)			
6-9	7.2 (6.0, 8.5)	16.8 (14.8, 18.7)	11.8 (10.2, 13.4)
10-14	4.9 (4.1, 5.8)	10.6 (8.3, 12.9)	6.6 (4.5, 8.7)
15-18	4.6 (2.7, 6.5)*	16.5 (14.8, 18.3)	10.3 (8.9, 11.6)
Ethnicity, n (%)			
Malay	4.7 (3.7, 5.7)	12.8 (11.4, 14.2)	7.7 (6.5, 8.8)
Indian	8.3 (5.8, 10.8)	21.0 (17.2, 24.9)	14.0 (10.7, 17.2)
Chinese	2.9 (1.9, 3.9)	10.5 (8.6, 12.4)	5.5 (4.1, 6.9)
Bumiputera/Orang Asli	2.7 (0.0, 5.5)	10.2 (4.5, 15.9)	7.3 (2.6, 12.0)
Other	3.1 (-0.5, 6.6)	13.2 (5.1, 21.3)	7.6 (1.7, 13.5)

Estimates are based on mixed effects Poisson regression models adjusted for ethnicity (apart from ethnicity-stratified models) and for clustering at the household level.

* Models were not adjusted for ethnicity in order to facilitate convergence.

**Table V:** Prevalence of overweight among children in Segamat, Malaysia according to 3 international BMI references

	Prevalence (95% CI)		
	WHO	IOTF	CDC
Overall	31.4 (30.0, 32.8)	26.5 (25.3, 27.8)	26.9 (25.7, 28.2)
Sex, n (%)			
Male	34.1 (32.1, 36.1)	28.4 (26.5, 30.2)	29.6 (27.6, 31.5)
Female	28.7 (26.8, 30.6)	24.8 (23.0, 26.5)	24.4 (22.7, 26.1)
Age, y, n (%)			
6-9	33.4 (30.7, 36.1)	27.5 (25.1, 30.0)	30.3 (27.8, 32.9)
10-14	36.2 (33.8, 38.6)	29.8 (27.7, 32.0)	30.3 (28.2, 32.5)
15-18	24.0 (21.9, 26.1)	21.8 (19.8, 23.8)	20.1 (18.1, 22.0)
Ethnicity, n (%)			
Malay	31.4 (29.7, 33.1)	26.6 (25.0, 28.1)	27.0 (25.5, 28.6)
Indian	28.1 (24.0, 32.3)	24.2 (20.3, 28.0)	24.0 (20.2, 27.8)
Chinese	33.9 (30.6, 37.2)	28.7 (25.6, 31.7)	29.2 (26.2, 32.3)
Bumiputera/Orang Asli	25.9 (16.6, 35.1)	21.6 (13.1, 30.0)	20.7 (12.4, 29.0)
Other	25.3 (13.9, 36.7)	18.7 (8.9, 28.4)	18.7 (8.9, 28.4)

Estimates are based on mixed effects Poisson regression models adjusted for ethnicity (apart from ethnicity-stratified models) and for clustering at the household level.

**Table VI:** Prevalence of obesity among children in Segamat, Malaysia according to 3 international BMI references

	Prevalence (95% CI)		
	WHO	IOTF	CDC
Overall	13.1 (11.8, 14.4)	9.1 (7.9, 10.2)	12.6 (11.3, 13.9)
Sex, n (%)			
Male	17.1 (15.7, 18.6)	11.4 (10.2, 12.6)	16.0 (14.6, 17.4)
Female	11.4 (9.4, 13.3)	8.6 (6.9, 10.4)	11.7 (9.7, 13.7)
Age, y, n (%)			
6-9	18.3 (16.3, 20.3)	13.9 (12.1, 15.6)	18.1 (16.1, 20.1)
10-14	16.1 (14.5, 17.6)	9.5 (8.3, 10.7)	14.6 (13.1, 16.1)
15-18	8.7 (6.3, 11.1)	7.8 (5.4, 10.2)	9.2 (6.6, 11.7)
Ethnicity, n (%)			
Malay	13.4 (12.0, 14.9)	9.3 (8.0, 10.6)	12.9 (11.4, 14.3)
Indian	11.1 (8.4, 13.8)	7.8 (5.5, 10.0)	10.3 (7.7, 12.9)
Chinese	12.8 (10.6, 15.0)	8.4 (6.6, 10.2)	12.4 (10.2, 14.5)
Bumiputera/Orang Asli	13.9 (7.2, 20.7)	12.7 (6.3, 19.1)	14.7 (7.8, 21.7)
Other	13.3 (5.2, 21.5)	8.1 (1.9, 14.4)	13.3 (5.1, 21.5)

Estimates are based on mixed effects Poisson regression models adjusted for ethnicity (apart from ethnicity-stratified models) and for clustering at the household level.

## Discussion

In this study of Malaysian children aged 6-18 years, we observed a high prevalence of overweight and obesity and a notable burden of underweight among children, regardless of the BMI reference used. Although there was good to excellent agreement between references when classifying child overweight or obesity, prevalence estimates were consistently higher for both when using the WHO reference, and lower for obesity when using the IOTF reference. There was poor to moderate agreement between the references when classifying child underweight, and prevalence estimates varied notably across all three. Our results suggest that the choice of BMI reference may notably influence the decision to offer clinical advice or management, and estimates regarding the health resources required to address malnutrition, in this and similar populations in low- and middle-income countries.

Our results are consistent with the double burden of malnutrition in this Malaysian population.^4^, ^9^ Although underweight and stunting have not been the focus of recent research, overweight and obesity among children is gaining recognition as a public health issue in Malaysia.^3^, ^4^, ^5^, ^30^, ^31^ However, there has been little consistency in the use of references and cut-offs in child malnutrition research and clinical practice guidelines in the country.^3^, ^4^, ^5^, ^6^, ^31^, ^32^ Our findings highlight the need for the consistent use of at least 1 reference and associated cut-offs across guidelines and studies to allow for streamlined clinical action and facilitate meaningful comparisons of malnutrition prevalence estimates across studies.

Most studies to date have not systematically compared both undernutrition and overnutrition classification using the 3 international references examined here. Some have compared older references previously recommended by the WHO and IOTF,^33^, ^34^, ^35^ many have assessed the WHO growth standards in children aged 0-5 years, including measures such as weight-for-height,^20^, ^36^, ^37^, ^38^, ^39^, ^40^, ^41^, ^42^ and yet others have also explored the comparability of locally constructed BMI references.^19^, ^21^, ^23^, ^34^, ^35^, ^43^, ^44^ Much of this evidence is based on European and American populations,^33^, ^34^, ^35^, ^36^, ^37^, ^38^, ^39^, ^40^, ^41^, ^43^, ^45^, ^46^ and research on the current references in Asian populations of wide age ranges has been limited.^19^, ^20^, ^21^, ^22^, ^23^, ^47^ Certain studies examining the references compared here have reported broadly similar patterns: agreement was greater between the IOTF and CDC references,^22^, ^48^, ^49^ the IOTF reference underestimated obesity prevalence compared with the other 2,^21^, ^22^, ^46^, ^49^ and overweight and obesity prevalence was generally higher and underweight prevalence lower when using the WHO reference.^20^, ^22^, ^48^, ^49^ Our study provides support for these trends and additionally suggests that differences between references in assessing BMI status are not always consistent between demographic subgroups.

An understanding of the comparability of the 3 anthropometric references is important in the context of their inherent limitations. Unlike adult anthropometric cut-offs, which are based on mortality or disease outcomes, cut-offs for children's anthropometric references are defined statistically (ie, based on deviation from the mean). Furthermore, the WHO and CDC references are based exclusively on data from children in the US, with no other populations represented.^15^, ^16^, ^17^ Finally, these references describe BMI distribution across age in all children from general populations, regardless of health status.^15^, ^16^, ^17^ Only the widely adopted WHO 2006 child growth standards provide prescriptive standards of how BMI should change in healthy children aged 0-5 years.^14^ These were based on the Multicenter Growth Reference Study, which found growth among healthy children in unconstrained environments to be very similar across 6 diverse populations.^18^, ^50^

In light of such limitations, multiple studies, including 1 from Malaysia, have reported the construction of anthropometric references based upon data collected from local populations.^22^, ^30^, ^44^, ^51^ Such studies argue that a universal reference cannot be applied to children from different populations because their growth patterns are too distinct.^30^ Yet, to our knowledge, there is no definitive evidence to support this claim. Results from the Multicenter Growth Reference Study imply the contrary for children less than 5 years of age,^18^, ^50^ and there is little evidence on the comparability of growth patterns of healthy older children living in unconstrained environments across diverse populations. Assessing the suitability of population-specific vs international references is difficult because there is no gold standard to compare against, and limited research has explored the utility of these references in predicting adverse outcomes in adulthood.^45^, ^52^ Methodologically, locally developed references may not always be constructed with as much rigor and/or statistical power as international references.^22^, ^30^, ^53^

Regardless, consensus on a single international reference is required to allow for accurate comparison of child malnutrition burden across studies worldwide.^14^ Among younger children, the WHO 2006 standards are suitable for this use.^14^, ^18^, ^50^ For older children, our results and other published data are less conclusive about the superiority of any one international reference.^54^ Apart from initial consensus on a single reference at present, longitudinal research examining adverse outcomes in adulthood is needed to assess the comparative predictive utility of these references among older children in diverse populations.^45^, ^52^ Given the recognition that measurement of optimal growth and development of prescriptive universal standards may be logistically highly difficult for older children,^16^, ^55^ such research is essential to inform the identification of the most suitable reference to effectively monitor malnutrition, or to clearly establish the potential need for improved references for this age group.

Importantly, the clinical diagnosis of malnutrition in a child should take into account other relevant factors additional to anthropometry, such as body composition, other signs of clinical undernutrition, or potential genetic syndromes.^56^ Similarly, high-level decisions to adopt a particular reference should be based on consultation with appropriate clinical, public health, and other experts, keeping in mind both setting-specific aspects and the general issues considered here.^14^

Despite standardization and training of staff in this study, the potential for measurement error remains because instruments were not calibrated during the survey. This may have some implications in terms of misclassification of anthropometry but is not expected to affect the relative differences and measures of agreement between references. Moreover, although patterns observed here were similar to previous evidence,^21^, ^22^, ^46^, ^48^, ^49^ our exact findings may not be fully generalizable to other populations. Nonetheless, our findings highlight the need for a better-informed, harmonized approach to assessing anthropometric status among older children across populations.

To conclude, we observed notable differences between 3 international BMI references in the classification of child underweight, overweight, and obesity, and subsequent estimates of prevalence. Our results indicate the need for the universal use of at least 1 reference to ensure comparability across populations, and for further longitudinal studies to assess the comparative ability of both international and local references to predict the future risk of cardiometabolic and other outcomes. Clearer consensus on the use of anthropometric references to measure malnutrition will be important to inform and guide global initiatives which drive national policy, particularly in low- and middle-income countries.^57^

## Figures and Tables

**Figure:**
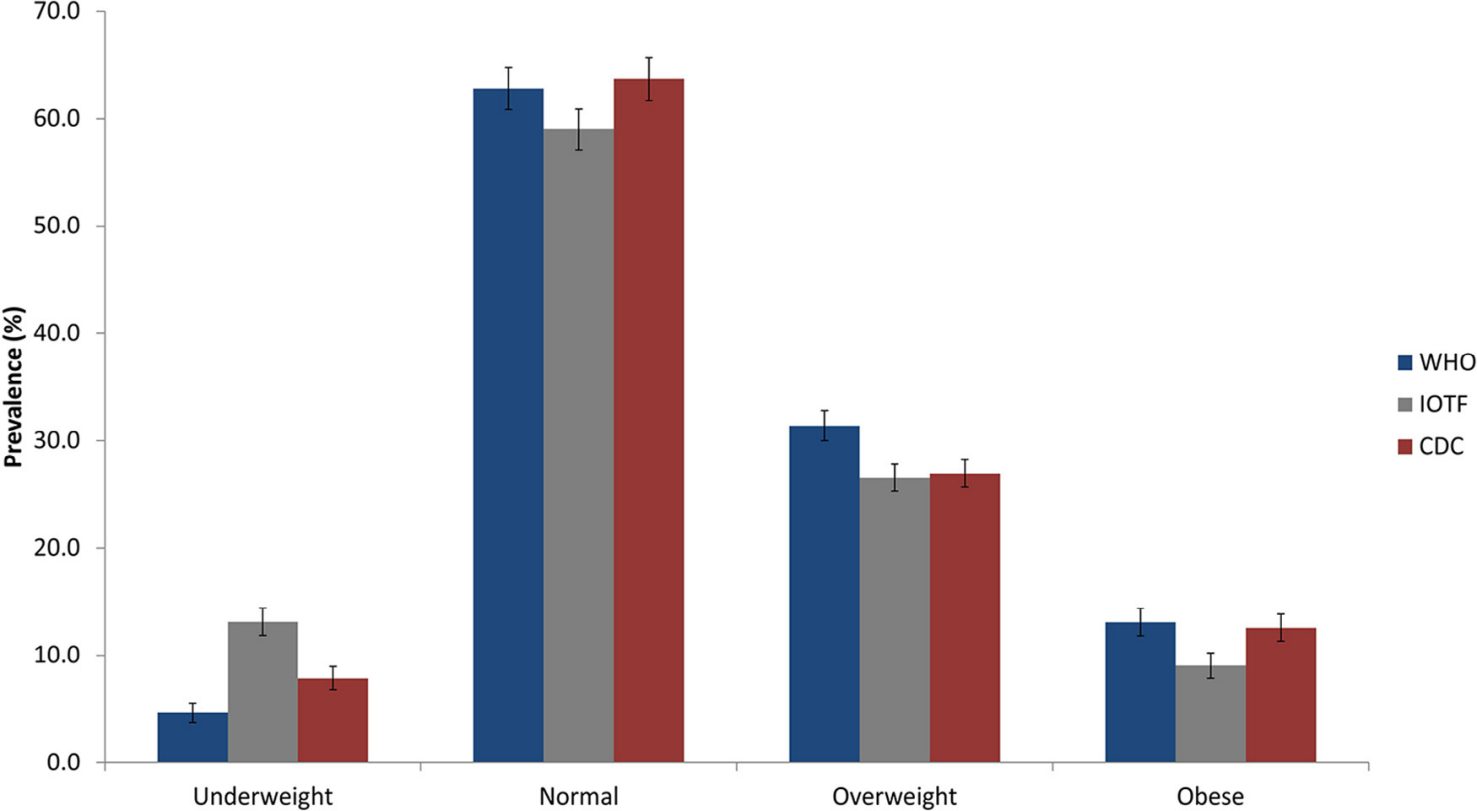
Overall prevalence of underweight, normal weight, overweight and obesity among children in Segamat, Malaysia according to 3 international references: CDC reference, IOTF reference, and WHO reference.

**Table II:** Relative classification of child BMI in accordance with 3 international BMI references (N = 6414)

	IOTF				CDC			
	Underweight	Normal	Overweight	Obese	Underweight	Normal	Overweight	Obese
WHO								
Underweight, n (%)	373 (100.0)	0 (0.0)	0 (0.0)	0 (0.0)	373 (100.0)	0 (0.0)	0 (0.0)	0 (0.0)
Normal, n (%)	551 (13.7)	3479 (86.3)	0 (0.0)	0 (0.0)	227 (5.6)	3803 (94.4)	0 (0.0)	0 (0.0)
Overweight, n (%)	0 (0.0)	308 (28.5)	772 (71.5)	0 (0.0)	0 (0.0)	284 (26.3)	784 (72.6)	12 (1.1)
Obese, n (%)	0 (0.0)	0 (0.0)	264 (28.4)	667 (71.6)	0 (0.0)	0 (0.0)	49 (5.3)	882 (94.7)
IOTF								
Underweight, n (%)					600 (64.9)	324 (35.1)	0 (0.0)	0 (0.0)
Normal, n (%)					0 (0.0)	3721 (98.3)	66 (1.7)	0 (0.0)
Overweight, n (%)					0 (0.0)	42 (4.1)	767 (74.0)	227 (21.9)
Obese, n (%)					0 (0.0)	0 (0.0)	0 (0.0)	667 (100.0)

**Table III:** Agreement between international references in classification of underweight, overweight, and obesity

	Underweight			Overweight			Obese		
	WHO-IOTF	WHO-CDC	IOTF-CDC	WHO-IOTF	WHO-CDC	IOTF-CDC	WHO-IOTF	WHO-CDC	IOTF-CDC
Overall	0.54	0.75	0.76	0.88	0.89	0.96	0.81	0.96	0.83
Sex									
Male	0.60	0.75	0.83	0.87	0.89	0.94	0.77	0.95	0.80
Female	0.49	0.75	0.70	0.90	0.89	0.97	0.87	0.98	0.87
Age (y)									
6-9	0.56	0.74	0.80	0.86	0.93	0.93	0.84	0.97	0.84
10-14	0.58	0.82	0.75	0.86	0.87	0.98	0.71	0.95	0.76
15-18	0.48	0.70	0.73	0.94	0.89	0.95	0.94	0.98	0.93
Ethnicity									
Malay	0.55	0.77	0.76	0.88	0.90	0.96	0.82	0.96	0.84
Indian	0.56	0.74	0.80	0.90	0.89	0.96	0.82	0.94	0.86
Chinese	0.45	0.70	0.70	0.88	0.89	0.94	0.78	0.97	0.80
Bumiputera/Orang Asli	0.44	0.55	0.86	0.88	0.86	0.92	0.97	0.97	0.93
Other	0.36	0.58	0.70	0.81	0.81	1.00	0.75	1.00	0.75

Agreement was calculated using the Cohen kappa coefficient.
